# Comparison of risk factor associations in UK Biobank against representative, general population based studies with conventional response rates: prospective cohort study and individual participant meta-analysis

**DOI:** 10.1136/bmj.m131

**Published:** 2020-02-12

**Authors:** G David Batty, Catharine R Gale, Mika Kivimäki, Ian J Deary, Steven Bell

**Affiliations:** 1 Department of Epidemiology and Public Health, University College London, London WC1E 6BT, UK; 2 School of Biological and Population Health Sciences, Oregon State University, Corvallis, OR, USA; 3 MRC Lifecourse Epidemiology Unit, University of Southampton, Southampton, UK; 4 Lothian Birth Cohorts, Department of Psychology, University of Edinburgh, Edinburgh, UK; 5 British Heart Foundation Cardiovascular Epidemiology Unit, Department of Public Health and Primary Care, University of Cambridge, Cambridge, UK; 6 National Institute for Health Research Blood and Transplant Unit in Donor Health and Genomics at the University of Cambridge, Cambridge, UK; 7 Stroke Research Group, Department of Clinical Neurosciences, University of Cambridge, Cambridge, UK

## Abstract

**Objective:**

To compare established associations between risk factors and mortality in UK Biobank, a study with an exceptionally low rate of response to its baseline survey, against those from representative studies that have conventional response rates.

**Design:**

Prospective cohort study alongside individual participant meta-analysis of other cohort studies.

**Setting:**

United Kingdom.

**Participants:**

Analytical sample of 499 701 people (response rate 5.5%) in analyses in UK Biobank; pooled data from the Health Surveys for England (HSE) and the Scottish Health Surveys (SHS), including 18 studies and 89 895 people (mean response rate 68%). Both study populations were linked to the same nationwide mortality registries, and the baseline age range was aligned at 40-69 years.

**Main outcome measure:**

Death from cardiovascular disease, selected malignancies, and suicide. To quantify the difference between hazard ratios in the two studies, a ratio of the hazard ratios was used with HSE-SHS as the referent.

**Results:**

Risk factor levels and mortality rates were typically more favourable in UK Biobank participants relative to the HSE-SHS consortium. For the associations between risk factors and mortality endpoints, however, close agreement was seen between studies. Based on 14 288 deaths during an average of 7.0 years of follow-up in UK Biobank and 7861 deaths over 10 years of mortality surveillance in HSE-SHS, for cardiovascular disease mortality, for instance, the age and sex adjusted hazard ratio for ever having smoked cigarettes (versus never) was 2.04 (95% confidence interval 1.87 to 2.24) in UK Biobank and 1.99 (1.78 to 2.23) in HSE-SHS, yielding a ratio of hazard ratios close to unity (1.02, 0.88 to 1.19). The overall pattern of agreement between studies was essentially unchanged when results were compared separately by sex and when baseline years and censoring dates were aligned.

**Conclusion:**

Despite a very low response rate, risk factor associations in the UK Biobank seem to be generalisable.

## Introduction

Well designed prospective cohort studies have considerable utility in identifying genetic and environmental risk factors for an array of somatic and psychiatric disorders. In the many contexts in which randomised controlled trials are not feasible owing to financial, ethical, or logistical constraints, this type of observational study provides the best approximation of causality. Although well phenotyped cohort studies have existed for decades, recent technological advances have led to low cost, high throughput methods to quantify genetic variation. Simultaneously expanding prospects for linkage to medical and non-medical electronic records has allowed construction of studies with the capacity to explore the effect of gene-environment combinations on health endpoints at a previously unheralded scale. Several countries have established such national “biobanks,”^1^ ^2^ are in the process of their formulation,^3^ ^4^ ^5^ or are planning such an endeavour.^6^

One such leading resource is UK Biobank, a prospective cohort study comprising around 500 000 middle aged and older people.^7^ Unusually in the context of medical research, baseline data have been open access since completion of curation in 2012,^8^ and, to date, the study has yielded in excess of 1000 publications.^9^ Although UK Biobank is rare in its combination of size and content, it also had an uncommonly low response to its baseline survey: of more than nine million people sent an invitation to participate, only around 6% did so.^10^ This achieved response rate was driven by the cost and time saving decision not to re-contact undecided potential participants.^11^ Presumably as a consequence, the project came in under budget and ahead of schedule.

Whereas such an approach is doubtless procedurally efficient, the long held view is that epidemiological studies need to achieve considerably higher response rates if their findings are to be credible.^12^ ^13^ Debates about the effect of non-response on estimations of chronic disease determinants in UK Biobank—its primary objective—and the wider necessity for representativeness have followed.^14^ ^15^ ^16^ ^17^ ^18^ ^19^ ^20^ ^21^ ^22^ ^23^ Despite more favourable baseline risk factor levels and mortality rates in UK Biobank relative to studies achieving a greater response,^24^ its principal investigators have consistently maintained that, because the exposures of interest have sufficient variance and the study sample is large, the generalisability of associations between risk factors and health outcomes is assured.^11^ ^25^ ^26^ Although findings from cohort studies sampled from highly select groups—Framingham residents and British civil servants,^27^ ^28^ among many others^29^—provide indirect support for this assertion, to our knowledge it has yet to be tested empirically.

To examine whether risk factor associations in UK Biobank are generalisable, in analyses of raw data from the study, we compared effect estimates for characteristics known to be linked to major causes of mortality against those from a pooling of data from nationally sampled cohort studies drawn from England and Scotland, all of which had a conventional response to their baseline surveys (mean 68%).^30^ With UK Biobank data being deployed across a range of scientific disciplines, we also chose an array of mortality endpoints and exposures. Given the nature of our research question, our focus was not on discovery of risk factors; rather, our aim was to test risk factor-endpoint associations that are well established on the basis of strong observational and/or experimental evidence. We therefore related demographic, social, behavioural, and biomedical risk factors to cardiovascular disease,^31^ ^32^ physical stature to cardiovascular disease and cancer,^33^ ^34^ ^35^ and educational attainment to suicide risk.^36^ ^37^ ^38^ ^39^

## Methods

We used individual level data from both UK Biobank,^7^ a prospective cohort study, and a pooling of 18 other prospective cohort studies with identical core protocols: the Health Survey for England (HSE; 15 studies)^40^ and the Scottish Health Surveys (SHS; three studies)^41^ (hereafter, HSE-SHS). The sampling and procedures of these studies have been well described.^42^ ^43^ In brief, baseline data collection in UK Biobank took place between 2006 and 2010 in 22 research assessment centres across the UK, resulting in a sample of 502 655 people aged 40 to 69 years (response rate 5.5%).^7^ In HSE and SHS, a total of 193 842 people aged 16-102 years (mean response rate 68%; range 58-93%^30^) participated in home based data collection between 1994 and 2008. For the purposes of this comparison, we restricted HSE-SHS data to the 89 895 people (48 364 women) with a baseline age range that matched UK Biobank. Participants in both studies gave informed consent.

### Assessment of baseline characteristics

In both UK Biobank and HSE-SHS, the following data were self-reported using identical or near identical enquiries: diagnosis by a physician of chronic disease (diabetes, hypertension, cardiovascular disease); use of multivitamins, lipid lowering drugs, blood glucose lowering drugs, and antihypertensive drugs; educational attainment; cohabitation status; and cigarette smoking habit. Although physical activity and alcohol intake were collected using somewhat different questions, we were able to derive comparable binary categories (current non-drinker versus the rest; physically inactive versus the rest).

During medical examinations, waist and hip circumference, as well as height and weight, were measured directly using standard protocols. Elevated waist:hip ratio was denoted by values of 0.90 or greater for men and 0.85 or greater for women^44^; obesity was indicated by a body mass index of 30 or above.^45^ Forced expiratory volume in one second, a measure of pulmonary function, was quantified using spirometry with the best of three (UK Biobank) or five (HSE-SHS) technically satisfactory exhalations used in our analyses. In UK Biobank, seated systolic and diastolic blood pressure measurements were made twice using the Omron HEM-7015IT digital blood pressure monitor (Omron Healthcare)^20^ or, exceptionally, a manual sphygmomanometer (6652 people); we used an average of the two readings. In HSE-SHS, three readings were taken using the Dinamap 8100 automated device,^46^ with a mean of the second and third values featuring in our analyses. We defined hypertension according to existing guidelines as systolic/diastolic blood pressure of 140/90 mm Hg or above, self-reported use of antihypertensive drugs, or both.^47^ Non-fasting venous blood was drawn in both studies.^48^ ^49^ Assaying took place at dedicated central laboratories for C reactive protein, glycated haemoglobin, and total cholesterol and high density lipoprotein cholesterol.^40^ ^48^

### Ascertainment of cause specific mortality

Participants in both studies were linked to mortality registries by using the procedures of the UK National Health Service Central Registry.^50^ We extracted underlying cause of death, coded according to ICD-10 (international classification of disease, 10th revision), from death certificate data.^50^ We generated the following mortality outcomes: cardiovascular disease, all cancers combined, lung cancer, smoking attributable cancers, obesity attributable cancers, and suicide. The ICD codes denoting these causes of death are given in supplemental table 1.

### Statistical analyses

We calculated hazard ratios and accompanying 95% confidence intervals by using Cox regression models,^51^ adjusting effect estimates for age and sex. In these survival analyses, we censored individuals according to the date of death or the end of follow-up (14 February 2011 in HSE, 31 December 2009 in SHS, 22 February 2016 for UK Biobank), whichever came first. To quantify the difference between the hazard ratios in each of the two studies, we calculated a ratio of the hazard ratio as we have done in other contexts^50^ (HSE-SHS was the referent). We used Stata version 15 for all analyses.

### Patient involvement

These analyses are based on existing data of typically healthy populations, and we were not involved in their recruitment. Thus, to our knowledge, no patients were explicitly engaged in designing the present research question or the outcome measures, nor were they involved in developing plans for recruitment, design, or implementation of the study. No patients were asked to advise on interpretation or writing up of results. Results from UK Biobank are routinely disseminated to study participants via the study website and social media outlets.

## Results

In table 1 (biomedical factors) and supplemental figure 1 (demographic, social, and behavioural factors plus drug use), we compare the baseline characteristics of participants in UK Biobank against those in the compilation of 18 cohort studies. UK Biobank study members were less likely to have had a sub-university level education, to be living alone or unmarried, to be sedentary, to have existing cardiovascular disease, or to be taking drug treatments for raised blood glucose, although the reverse was seen for lipid lowering and antihypertensive drugs. In analyses restricted to study members not reporting the use of such therapies, we essentially observed no marked difference between studies members for total and high density lipoprotein cholesterol or for glycated haemoglobin. Whereas values for C reactive protein were lower in UK Biobank, both systolic and diastolic blood pressure were somewhat higher. Taken together, UK Biobank participants had a generally more favourable risk factor profile.

**Table 1:** Summary of baseline biomedical characteristics in UK Biobank and Health Survey for England and Scottish Health Surveys (HSE-SHS) cohort studies

Characteristics	UK Biobank	HSE-SHS
No of studies	1	18
No of participants (women)	502 655 (273 472)	89 895 (48 364)
Mean (SD) age, years	56.5 (8.10)	53.5 (8.6)
Mean (SD) FEV_1_, L	2.81 (0.80)	2.89 (0.89)
Mean (SD) total cholesterol, mmol/L	5.89 (1.07)	5.95 (1.14)
Median (IQR) high density lipoprotein cholesterol, mmol/L	1.43 (1.20-1.71)	1.40 (1.20-1.70)
Median (IQR) glycated haemoglobin, mmol/mol	35.0 (32.6-37.4)	36.6 (33.3-40.9)
Median (IQR) C reactive protein, mg/L	1.26 (0.63-2.49)	1.50 (0.70-3.10)
Mean (SD) systolic blood pressure, mm Hg	137.7 (19.3)	133.3 (18.4)
Mean (SD) diastolic blood pressure, mm Hg	81.6 (10.6)	76.6 (11.5)

FEV_1_=forced expiratory volume in one second; IQR=interquartile range; SD=standard deviation.

Sample size given is for study members with age data only and is lower for all other characteristics. Analyses for total cholesterol and high density lipoprotein exclude participants taking lipid lowering drugs; analyses for glycated haemoglobin exclude people with self-reported diabetes and those taking blood glucose lowering drugs; analyses for C reactive protein exclude people with values >10 mg/L; and analyses for blood pressure exclude people taking antihypertensive drugs.

In UK Biobank, 14 288 deaths from all causes occurred during an average of 7.0 years of follow-up in 499 701 people who consented to be linked to mortality registers. In the combined HSE-SHS databases, 10 years of mortality surveillance gave rise to 7861 deaths in 89 895 people with these consents. Of the five mortality categories examined in survival analyses, rates of cardiovascular disease, all cancers combined, and tobacco and obesity attributable cancers were markedly lower in UK Biobank, whereas the rate of suicide was higher (supplemental table 2).

**Fig 1:**
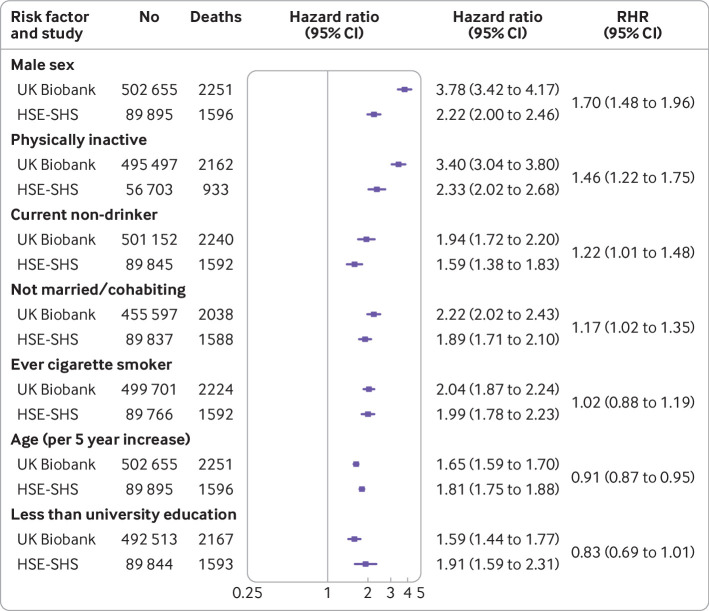
Association of baseline demographic and behavioural characteristics with cardiovascular disease mortality in UK Biobank and Health Survey for England/Scottish Health Surveys (HSE-SHS) cohort studies. Hazard ratios are adjusted for age and sex, with the exception of individual effects for age and sex which are mutually adjusted. Squares indicate hazard ratios and error bars denote 95% CI for relation of each characteristic with risk of death outcome. Ratio of hazard ratios (RHR) summarises between study differences (HSE-SHS is reference group) for effect estimates for each outcome

**Fig 2:**
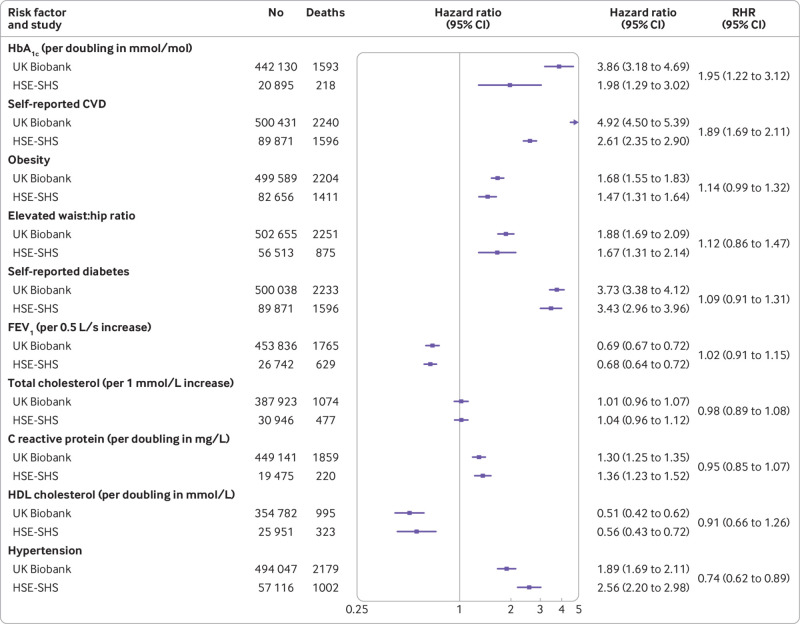
Association of baseline biomedical characteristics with cardiovascular disease (CVD) mortality in UK Biobank and Health Survey for England/Scottish Health Surveys (HSE-SHS) cohort studies. Hazard ratios are adjusted for age and sex. Squares indicate hazard ratios and error bars denote 95% CI for relation of each characteristic with risk of death outcome. Ratio of hazard ratios (RHR) summarises between study differences (HSE-SHS is reference group) for effect estimates for each outcome. Distributions of glycated haemoglobin (HbA_1c_), C reactive protein, and high density lipoprotein (HDL) cholesterol were skewed, so they were log_2_ transformed and effect estimates reflect doubling for each biomarker. Elevated waist:hip ratio was denoted by ≥0.90 for men and ≥0.85 for women; obesity was indicated by body mass index ≥30. FEV_1_=forced expiratory volume in one second

In figure 1, for each study, we depict the association of known baseline demographic and behavioural risk factors with cardiovascular disease mortality. The expected direction of association was the same in both studies for the seven characteristics, whereby being male, being of higher age, being physically inactive, not drinking alcohol, not being married or cohabiting, being a current or former smoker, and not having a higher education degree were related to elevated rates of cardiovascular disease mortality. Some modest differences existed in the magnitude of these effects in four of the risk factors examined, such that hazard ratios were typically higher in UK Biobank. When we explored the links between biomedical factors and cardiovascular disease mortality (fig 2), all 10 of the biomarkers featured showed known associations with cardiovascular disease deaths in both studies. Although agreement between studies was again high, some heterogeneity was also apparent in the strength of these effects for higher levels of glycated haemoglobin, existing cardiovascular disease (stronger effects in UK Biobank than in HSE-SHS for both risk factors), and hypertension (the reverse). Taken together, a high degree of concordance existed for cardiovascular disease risk factor associations in UK Biobank and HSE-SHS.

**Fig 3:**
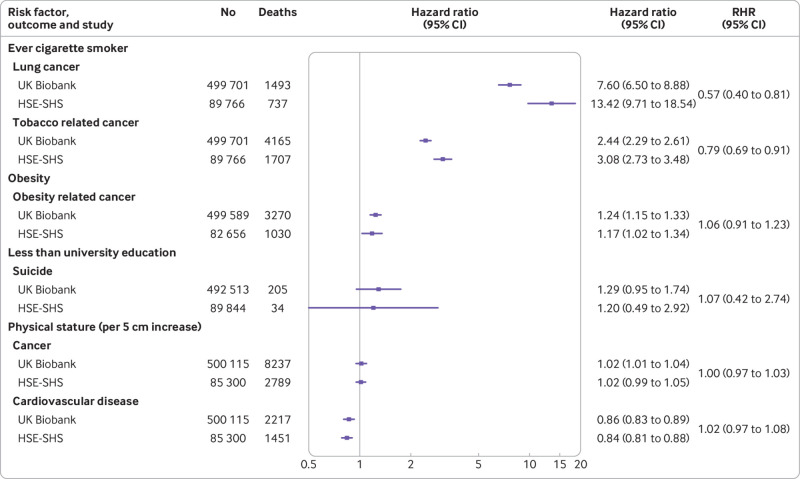
Association of selected baseline characteristics with cause specific mortality in UK Biobank and Health Survey for England/Scottish Health Surveys (HSE-SHS) cohort studies. Hazard ratios are adjusted for age and sex. Squares indicate hazard ratios and error bars denote 95% CI for relation of each characteristic with risk of death outcome. Ratio of hazard ratios (RHR) summarises between study differences (HSE-SHS is reference group) for effect estimates for each outcome

Next, we examined the association of selected baseline factors with some non-cardiovascular disease mortality outcomes, including different presentations of cancer deaths and completed suicides (fig 3). Known risk factor associations were replicated across both studies. The magnitude of the association of cigarette smoking with lung cancer and malignancies causatively linked to tobacco intake were weaker for UK Biobank, whereas obesity and cancers attributed to it yielded similar effects in each study. Hazard ratios were also essentially the same for lower educational attainment and suicide, although statistical power was modest in these analyses, particularly for HSE-SHS, as evidenced by the wide confidence intervals. Physical stature showed the predicted opposing and shallow gradients for cardiovascular disease (negative) and cancer (positive); again, effect sizes were very similar in both studies.

Given well known secular changes in risk factors levels, as evidenced by repeat cross sectional surveys,^52^ we used sensitivity analyses to explore the effect of having the same calendar period (2006-08) for recruitment of participants in HSE and UK Biobank (supplemental figure 2), and in another set of analyses we additionally aligned mortality surveillance by right censoring in UK Biobank (follow-up to 14 February 2011) (supplemental figure 3). Owing to a rarity of events, these analyses were restricted to death from cardiovascular disease. Risk factor associations were essentially the same as those apparent in the main analyses, the only exception being obesity. We also found that results held in sex specific analyses for demographic and behavioural characteristics (supplemental figure 4) and biomarkers (supplemental figure 5). Lastly, given that, as described, the self reported use of drugs for lowering blood pressure and lipids was higher in members of UK Biobank relative to our comparator cohorts, we tested whether this was also evident for other health seeking behaviours such as vitamin and mineral supplementation. The prevalence of such use was counter to expectations, being lower in UK Biobank (21.8%) than in HSE-SHS (33.1%).

## Discussion

In a comparison of findings between UK Biobank and 18 studies from the HSE-SHS consortium, we found close agreement for a series of well established risk factors for cause specific mortality. These concordant results were apparent despite the response rate in UK Biobank being an order of magnitude lower than in the comparator cohorts and that study having a generally more favourable prevalence of sociodemographic, behavioural, and health related characteristics at baseline and lower rates of cause specific mortality during follow-up, as shown here and elsewhere.^24^

### Findings from other studies

The only other analyses of risk factor relations in UK Biobank versus those in comparator studies of which we are aware are those for cardiometabolic multimorbidity and venous thromboembolism in the Emerging Risk Factors Collaboration, a pooling of data from up to 91 cohort studies.^53^ ^54^ The goal of those papers, however, was discovery of risk factors rather than testing well established associations between risk factors and chronic disease. Blood based biomarkers in UK Biobank were also not available at the time of these analyses and, in the report featuring venous thromboembolism as the endpoint of interest,^54^ inter-study comparison was hampered by differing approaches to disease ascertainment.

As described, UK Biobank principal investigators, while acknowledging that their study has little value in describing the prevalence of a risk factor or rates of mortality—never stated objectives—have attempted to minimise unease around the investigation of chronic disease aetiology—its primary purpose—by arguing that generalisable associations with risk factors can be obtained in non-representative samples provided sufficiently large numbers of people with a range of exposures are included.^11^ ^25^ ^26^ They cite the circumstantial evidence of cohort studies drawing on selected populations that have markedly higher response rates than UK Biobank—Framingham residents,^27^ British physicians,^55^ US nurses^56^—all of which produced results that have subsequently been shown to be transportable to general population based studies and have contributed much to the prevention of cardiovascular disease and selected cancers. Similarly, our findings mirror those from analyses in which we have compared risk factors for coronary heart disease in another highly select group, a cohort of British civil servants (the Whitehall II prospective cohort study), with those from a cohort based on the general population (the British Regional Heart Study).^57^ In those analyses, we also found near identical risk factor relations across studies.

### Limitations of study

Our work inevitably has some shortcomings. Firstly, whereas UK Biobank includes people from the contiguous countries that comprise the UK, the comparator studies included no data from Wales. We have no reason to believe that the absence of these data would affect our results, however. Secondly, whereas core elements of data collection in the HSE-SHS consortium were essentially constant across studies, scientific themes for data collection differed from year to year.^40^ As such, selected biomedical data were not collected in all survey years and the analytical sample size was diminished as a result. Thirdly, for two variables—physical activity and alcohol intake—baseline data were not directly comparable between studies, although we were able to harmonise data into binary groups. These represent two of 23 risk factor-outcome combinations, however, which means that exclusion of these data would have no effect on our overall conclusions of high agreement between studies. Fourthly, the mode of data collection differed between studies—data collection in UK Biobank took place in designated research centres, whereas it was home based in HSE-SHS—although we see no strong justification for this affecting our results. Fifthly, in the main analyses, the endpoint of the interest was cardiovascular disease mortality, which is an amalgam of both incidence of the condition and survival with it. This raises the question of whether risk factor effects differ for incidence, which is temporally closer to assessment of exposure than is death. However, comparison of risk factors for coronary heart disease and stroke, as ascertained from mortality records and hospital admissions (incidence), have shown no evidence of differential associations.^58^ ^59^ Lastly, although blood samples have been frozen in HSE-SHS, so offering the potential for later genome sequencing, comparison with genetic risk prediction of chronic disease in UK Biobank is currently not possible. From a purely gene-outcome association perspective, however, with genetic variants being unlikely to be associated with either self-selection into the study or confounding factors, UK Biobank is likely to produce generalisable estimates of genetic risk.^19^

### Conclusions

Despite a low response rate, risk factor associations in UK Biobank seem to be generalisable. This suggests that the cost and time saving features of recruitment of study members did not affect aetiological utility.

## What is already known on this topic

The primary objective of UK Biobank is to identify risk factors for chronic diseases and injuries of public health importance
That the baseline response rate was an order of magnitude lower than is conventional has led to debate as to the generalisability of its findings
Relative to studies with higher response rates and national statistics, baseline risk factor profile and mortality rates in UK Biobank are more favourable, but the impact, if any, on risk factor associations is unknown

## What this study adds

This is the first study to directly compare risk factor associations in UK Biobank with nationally representative cohort studies with conventional response rates
Associations of a wide range of risk factors with mortality outcomes showed close agreement between studies
Risk factor associations in UK Biobank seem to be generalisable

## Web extra

Extra material supplied by authors

## Notes

Contributions: GDB generated the idea for the study, formulated an analytical plan, and wrote the manuscript. CRG (UK Biobank) and SB (HSE-SHS) formulated an analytical plan and did all the data analyses. SB prepared the figures. All authors commented on an earlier version of the manuscript. The corresponding author attests that all listed authors meet authorship criteria and that no others meeting the criteria have been omitted. CRG had full access to UK Biobank data, and SB had full access to HSE-SHS data. GDB takes responsibility for the decision to submit the manuscript for publication. GDB, CRG, and SB are the guarantors.

Ethical approval: In UK Biobank, ethical approval for data collection was received from the North-West Multi-centre Research Ethics Committee and the research was carried out in accordance with the Declaration of Helsinki of the World Medical Association. In HSE-SHS, ethical approval for data collection was granted by the London Research Ethics Council or local research ethics councils. No additional ethical approval was required for the analyses of the data. Participants in both studies gave informed consent.

Data sharing: Data from UK Biobank (https://www.ukbiobank.ac.uk/) and the Health Surveys for England and the Scottish Health Surveys (https://data-archive.ac.uk/) are available to bona fide researchers on application. Part of this research has been conducted using the UK Biobank Resource under Application 10279.

Transparency: GDB affirms that the manuscript is an honest, accurate, and transparent account of the study being reported; that no important aspects of the study have been omitted; and that any discrepancies from the study as planned (and, if relevant, registered) have been explained.

Dissemination to participants and related patient and public communities: Findings will be disseminated via the media departments of the authors’ institutes. Results from UK Biobank are routinely disseminated to study participants via the study website and Twitter feed.

Pre-print deposition: medRxiv (https://www.medrxiv.org/content/10.1101/19004705v1).
