# Supplementary material for: Comparison of risk factor associations in UK Biobank against representative, general population based studies with conventional response rates: prospective cohort study and individual participant meta-analysis
Source: BMJ. 2020 Feb 12;368:m131. doi: 10.1136/bmj.m131 (PMC7190071; doi:10.1136/bmj.m131)
Supplement: Supplementary file 1 — Supplementary materials [file batd052480.ww1.pdf]

Supplemental Table 1. Mortality outcomes in the present analyses

Mortality outcome	ICD 10 codes
All cancers combined	C00-C97
Obesity-attributable cancers[1]	Breast (postmenopausal) C50; Colon C18; Rectum C19-20; Pancreas C25; Endometrium C54; Kidney C64; Ovary C56 & C57.0–4; Multiple myeloma C90; Liver C22; Thyroid C73; Gallbladder C23-24; Oesophageal adenocarcinoma C15 & morphology 8140–8141, 8143–8145, 8190–8231, 8260–8263, 8310,8401, 8480–8490, 8550–8551, 8570–8574, 8576; and Gastric cardia C16.0
Smoking-attributable cancers[2,3]	Lip, oral cavity, pharynx C00–C14, oesophagus C15, stomach C16, colon and rectum C18–C20, liver C22, pancreas C25, larynx C32, trachea, lung, bronchus C33–C34, cervix uteri C53, kidney and renal pelvis C64–C65, urinary bladder C67, and acute myeloid leukemia C92.0
Cardiovascular disease	I20-5, I50, I60-70, I73, I74
Suicide[4]	Suicide and self-inflicted poisoning by solid or liquid substances (E950-E959), injury undetermined whether accidentally or purposely inflicted (E980-E989), terrorism (U03.1 and U03.9), intentional self-harm (X60-X84), event of undetermined intent (Y10-Y34), sequelae of intentional self-harm, assault and events of undetermined intent (Y87), and sequelae of unspecified external cause (Y89.9))
Lung cancer	C34

Supplemental Table 2. Mortality rates in participants in UK Biobank and the HSE-SHS cohort studies

	UKBB	HSE-SHS
Number of adults (women)	502,655 (273,472)	89,895 (48,364)
Duration of mortality surveillance (years, mean [SD])	6.99 (1.03)	9.62 (4.39)
Total number of deaths	14,288	7,861
Mortality rate for cardiovascular disease	64	185
Mortality rate for all cancers combined	236	342
Mortality rate for tobacco-attributable cancers	120	198
Mortality rate for obesity-attributable cancers	94	132
Mortality rate for suicide	6	4

Mortality rates are expressed per 100,000 person-years. SD, standard deviation.

Supplemental Figure 1. Prevalence of baseline characteristics in UK Biobank and the HSE-SHS cohort studies

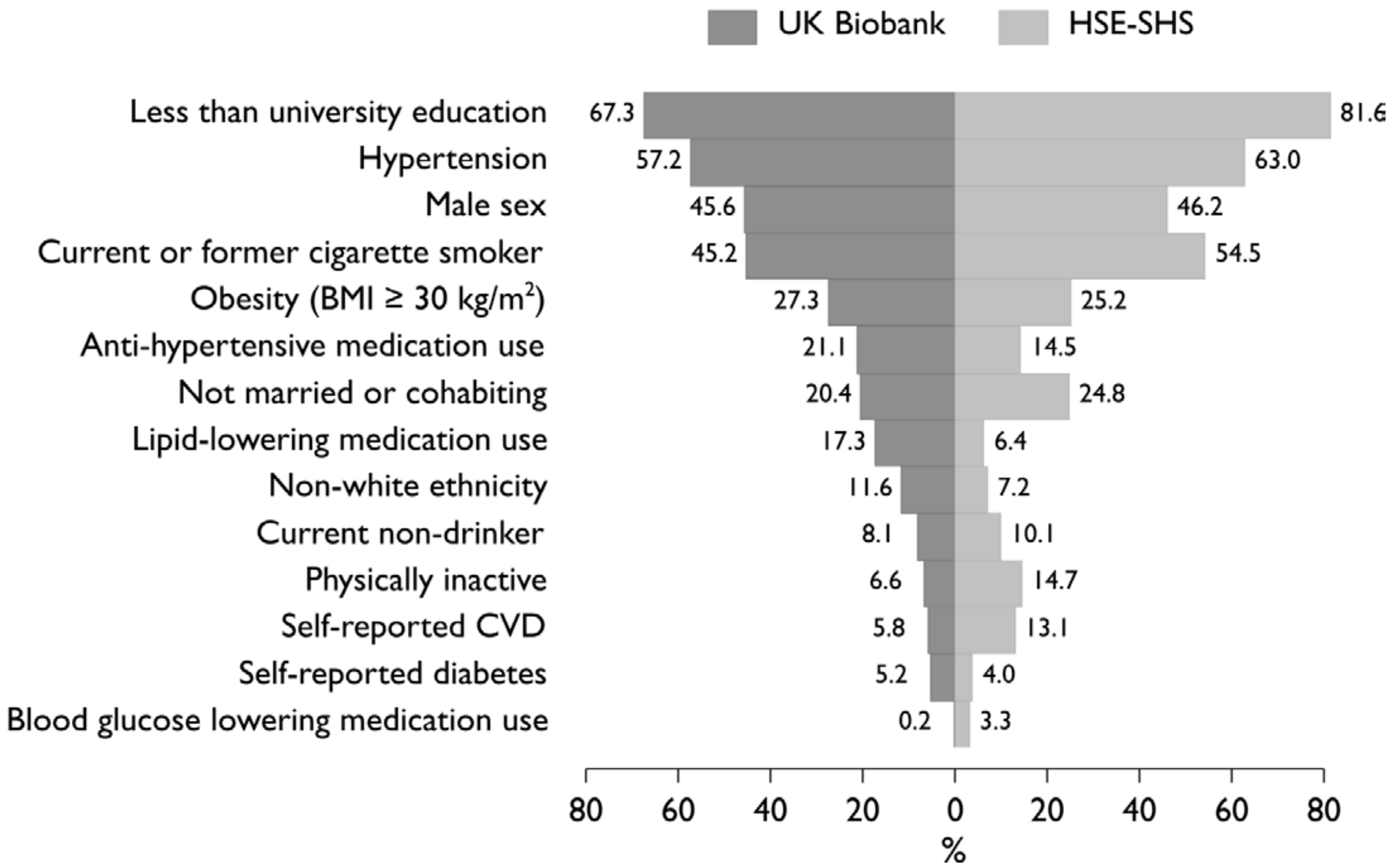

Selected comparisons have been made by Fry and others[5] using published findings from four HSE studies (2006, 2008, 2009, and 2010). Our analyses are based on analyses of raw data from 18 cohort studies from England and Scotland which include two of those HSE studies (2006, 2008). Analyses comprise 502,655 people in UK Biobank and 89,895 in HSE-SHS. BMI, body mass index; CVD, cardiovascular disease.

Supplemental Figure 2. Association of selected baseline characteristics with cardiovascular disease mortality in UK Biobank and the HSE cohort studies – analyses based on the same baseline survey years (2006-8)

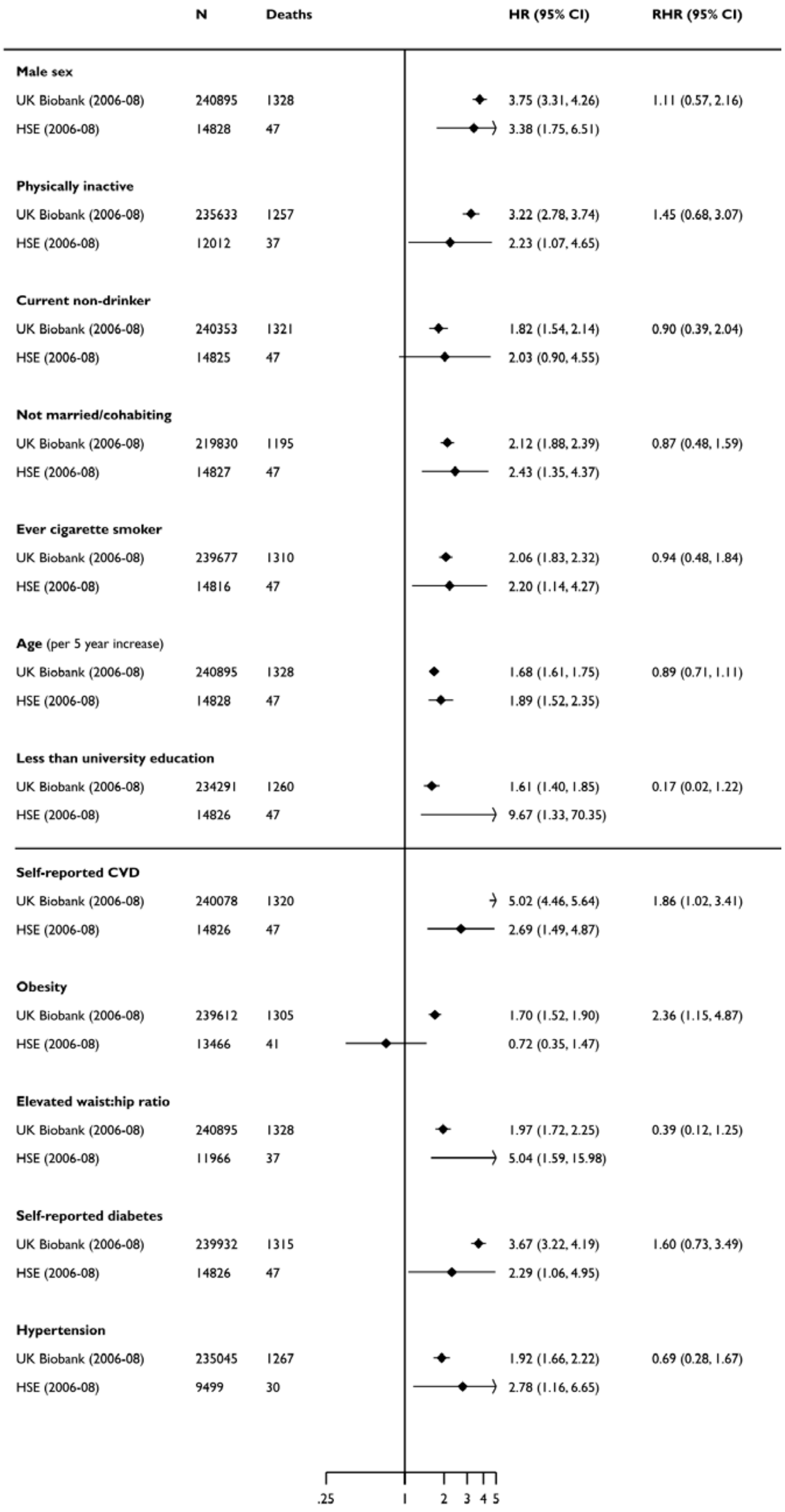

Hazard ratios are age- and sex-adjusted with the exception of age and sex which are mutually adjusted. The shaded diamonds indicate the hazard ratio (HR) and error bars denote the 95% confidence interval (CI) for the relation of each characteristics with the risk of death outcome. The ratio of hazard ratio (RHR) summarises the difference (HSE-SHS is the referent group) between that effect estimates for the outcome. CVD, cardiovascular disease.

Supplemental Figure 3. Association of selected baseline characteristics with cardiovascular disease mortality in UK Biobank and the HSE cohort studies – analyses based on the same baseline survey years (2006-8) and censoring date (February 2011)

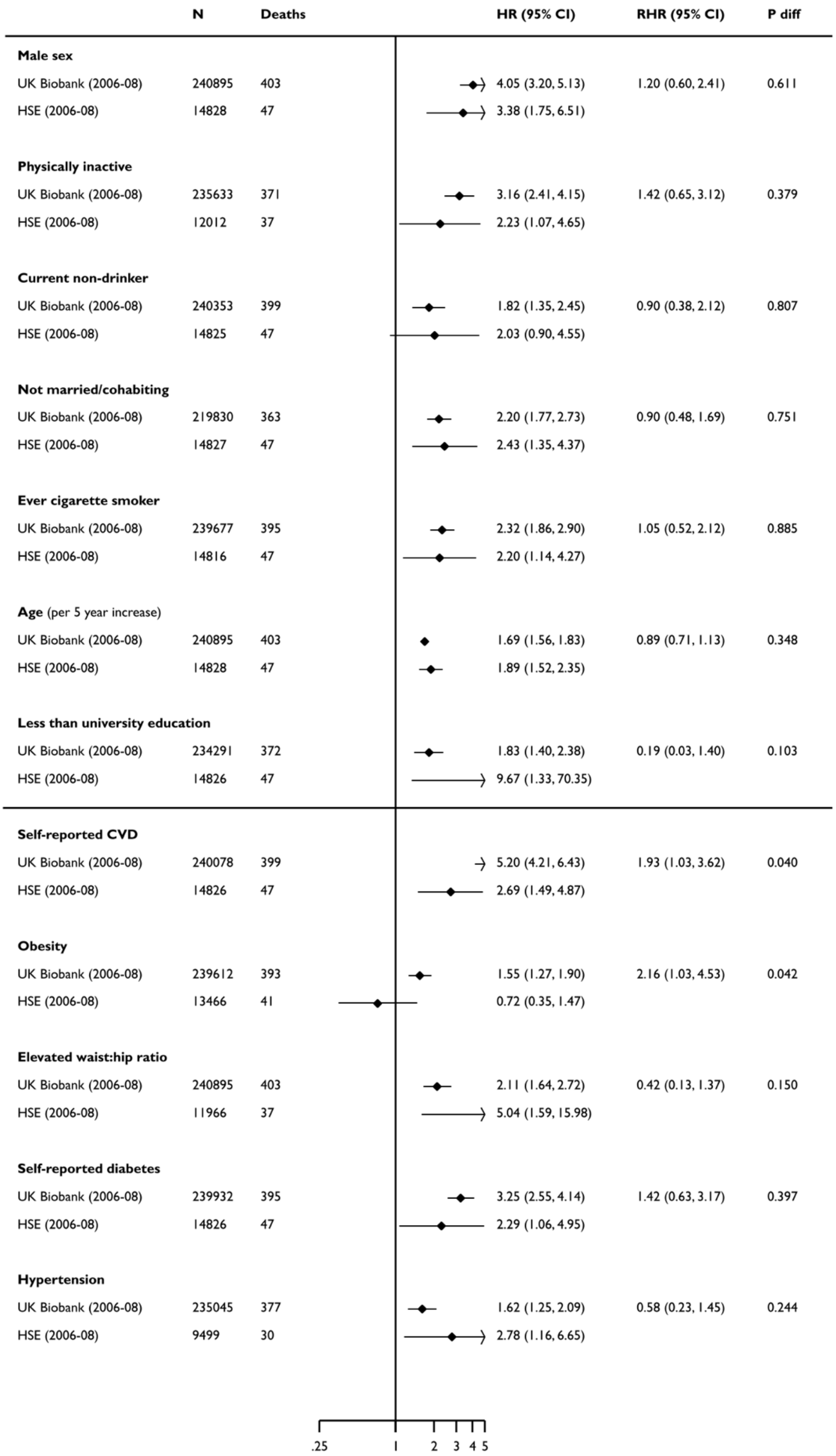

Hazard ratios are age- and sex-adjusted with the exception of age and sex which are mutually adjusted. The shaded diamonds indicate the hazard ratio (HR) and error bars denote the 95% confidence interval (CI) for the relation of each characteristics with the risk of death outcome. The ratio of hazard ratio (RHR) summarises the difference (HSE-SHS is the referent group) between that effect estimates for the outcome. CVD, cardiovascular disease.

Supplemental Figure 4. Association of baseline demographic and behavioural characteristics with cardiovascular disease mortality in UK Biobank and the HSE-SHS cohort studies – stratification by gender

Men

	N	Deaths	HR (95% CI)	RHR (95% CI)
**Physically inactive**				
UK Biobank	225912	1655	3.40 (2.99, 3.88)	1.52 (1.15, 2.00)
HSE-SHS	30648	340	2.24 (1.76, 2.86)	
**Current non-drinker**				
UK Biobank	228421	1716	1.81 (1.55, 2.11)	1.03 (0.80, 1.32)
HSE-SHS	48332	563	1.76 (1.44, 2.15)	
**Not married/cohabiting**				
UK Biobank	213476	1573	2.43 (2.19, 2.70)	1.37 (1.13, 1.67)
HSE-SHS	48333	563	1.77 (1.50, 2.09)	
**Ever cigarette smoker**				
UK Biobank	227749	1704	2.06 (1.85, 2.29)	1.03 (0.84, 1.27)
HSE-SHS	48319	564	1.99 (1.67, 2.36)	
**Age** (per 5 year increase)				
UK Biobank	229183	1724	1.60 (1.54, 1.66)	0.83 (0.77, 0.89)
HSE-SHS	48364	566	1.93 (1.81, 2.05)	
**Less than university education**				
UK Biobank	224391	1659	1.54 (1.37, 1.72)	0.73 (0.50, 1.08)
HSE-SHS	48337	565	2.09 (1.45, 3.01)	

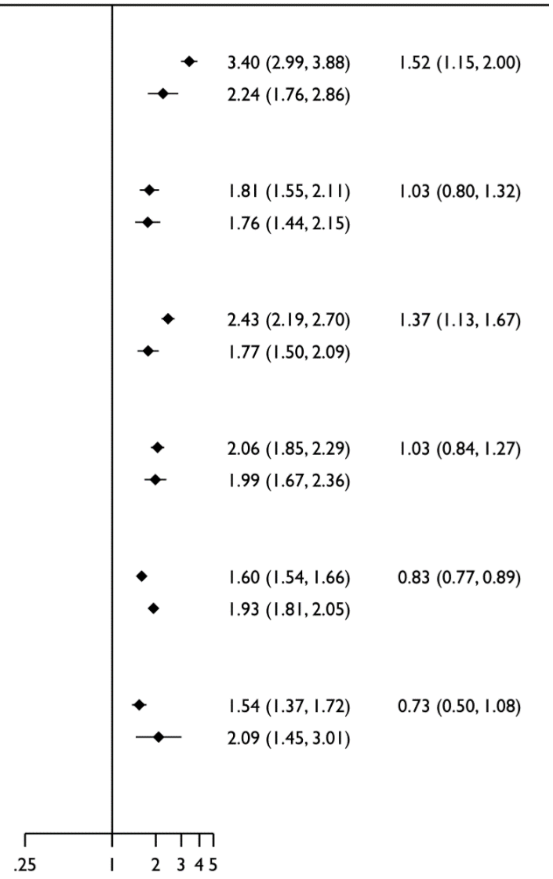

Women

	N	Deaths	HR (95% CI)	RHR (95% CI)
**Physically inactive**				
UK Biobank	269585	507	3.34 (2.69, 4.15)	1.42 (1.07, 1.87)
HSE-SHS	26055	593	2.36 (1.99, 2.80)	
**Current non-drinker**				
UK Biobank	272731	524	2.21 (1.79, 2.73)	1.55 (1.16, 2.08)
HSE-SHS	41513	1029	1.42 (1.16, 1.74)	
**Not married/cohabiting**				
UK Biobank	242121	465	1.61 (1.33, 1.95)	0.82 (0.65, 1.04)
HSE-SHS	41504	1025	1.96 (1.72, 2.24)	
**Ever cigarette smoker**				
UK Biobank	271952	520	2.06 (1.73, 2.45)	1.02 (0.81, 1.28)
HSE-SHS	41447	1028	2.02 (1.74, 2.35)	
**Age** (per 5 year increase)				
UK Biobank	273472	527	1.83 (1.70, 1.97)	1.04 (0.96, 1.13)
HSE-SHS	41531	1030	1.76 (1.68, 1.83)	
**Less than university education**				
UK Biobank	268122	508	1.82 (1.44, 2.32)	0.98 (0.71, 1.36)
HSE-SHS	41507	1028	1.86 (1.49, 2.32)	

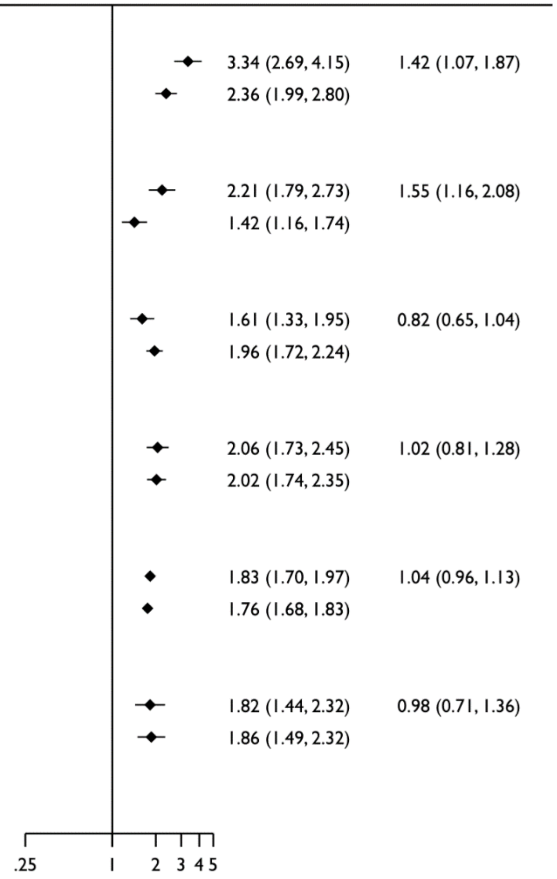

Hazard ratios are age- and sex-adjusted. The shaded diamonds indicate the hazard ratio (HR) and error bars denote the 95% confidence interval (CI) for the relation of each characteristics with the risk of death outcome. The ratio of hazard ratio (RHR) summarises the difference (HSE-SHS is the referent group) between that effect estimates for the outcome.

Supplemental Figure 5. Association of baseline biomedical characteristics with cardiovascular disease mortality in UK Biobank and the HSE-SHS cohort studies – stratification by gender

Men

	N	Deaths	HR (95% CI)	RHR (95% CI)
**HbA1c** (per doubling in mmol/mol)				
UK Biobank	198688	1191	3.94 (3.20, 4.85)	1.01 (0.49, 2.09)
HSE-SHS	11200	78	3.90 (1.94, 7.82)	
**Self-reported CVD**				
UK Biobank	228109	1714	4.90 (4.43, 5.42)	1.93 (1.57, 2.37)
HSE-SHS	48352	566	2.54 (2.12, 3.03)	
**Obesity**				
UK Biobank	227562	1689	1.76 (1.60, 1.94)	1.32 (1.07, 1.63)
HSE-SHS	44206	500	1.33 (1.10, 1.60)	
**Elevated waist:hip ratio**				
UK Biobank	229183	1724	1.89 (1.66, 2.15)	1.12 (0.82, 1.54)
HSE-SHS	30317	298	1.68 (1.26, 2.24)	
**Self-reported diabetes**				
UK Biobank	227770	1710	3.55 (3.18, 3.97)	0.71 (0.54, 0.92)
HSE-SHS	48352	566	5.02 (3.97, 6.36)	
**FEV1** (per 0.5 litres/second)				
UK Biobank	205164	1345	0.70 (0.68, 0.73)	1.23 (1.08, 1.41)
HSE-SHS	14171	212	0.57 (0.50, 0.64)	
**Total cholesterol** (per 1 mmol/L increase)				
UK Biobank	165632	767	0.99 (0.92, 1.06)	0.91 (0.78, 1.05)
HSE-SHS	16680	165	1.09 (0.96, 1.25)	
**C-reactive protein** (per doubling in mg/L)				
UK Biobank	206473	1419	1.34 (1.29, 1.40)	1.03 (0.85, 1.25)
HSE-SHS	10261	68	1.30 (1.07, 1.57)	
**HDL-cholesterol** (per doubling in mmol/L)				
UK Biobank	152947	719	0.58 (0.47, 0.72)	1.33 (0.82, 2.17)
HSE-SHS	14071	107	0.43 (0.28, 0.67)	
**Hypertension**				
UK Biobank	224588	1656	1.89 (1.65, 2.15)	0.64 (0.47, 0.86)
HSE-SHS	30881	345	2.95 (2.26, 3.86)	

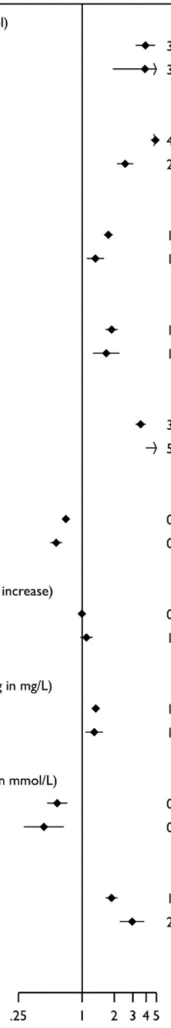

Women

	N	Deaths	HR (95% CI)	RHR (95% CI)
**HbA1c** (per doubling in mmol/mol)				
UK Biobank	243442	402	3.28 (1.93, 5.55)	2.16 (1.00, 4.63)
HSE-SHS	9695	140	1.52 (0.87, 2.64)	
**Self-reported CVD**				
UK Biobank	272322	526	5.25 (4.26, 6.48)	1.98 (1.55, 2.53)
HSE-SHS	41519	1030	2.65 (2.33, 3.02)	
**Obesity**				
UK Biobank	272027	515	1.43 (1.20, 1.72)	0.92 (0.73, 1.16)
HSE-SHS	38450	911	1.56 (1.35, 1.79)	
**Elevated waist:hip ratio**				
UK Biobank	273472	527	1.85 (1.55, 2.19)	1.21 (0.76, 1.94)
HSE-SHS	26196	577	1.52 (0.98, 2.36)	
**Self-reported diabetes**				
UK Biobank	272268	523	4.73 (3.79, 5.89)	1.66 (1.25, 2.21)
HSE-SHS	41519	1030	2.85 (2.37, 3.42)	
**FEV1** (per 0.5 litres/second)				
UK Biobank	248672	420	0.64 (0.59, 0.71)	0.90 (0.80, 1.02)
HSE-SHS	12571	417	0.71 (0.67, 0.75)	
**Total cholesterol** (per 1 mmol/L increase)				
UK Biobank	222291	307	1.04 (0.93, 1.15)	1.04 (0.89, 1.20)
HSE-SHS	14266	312	1.00 (0.91, 1.11)	
**C-reactive protein** (per doubling in mg/L)				
UK Biobank	242668	440	1.19 (1.10, 1.27)	0.85 (0.73, 0.98)
HSE-SHS	9214	152	1.40 (1.23, 1.60)	
**HDL-cholesterol** (per doubling in mmol/L)				
UK Biobank	201835	276	0.37 (0.26, 0.52)	0.55 (0.34, 0.88)
HSE-SHS	11880	216	0.67 (0.48, 0.92)	
**Hypertension**				
UK Biobank	269455	523	1.85 (1.51, 2.28)	0.78 (0.60, 1.03)
HSE-SHS	26235	657	2.36 (1.97, 2.84)	

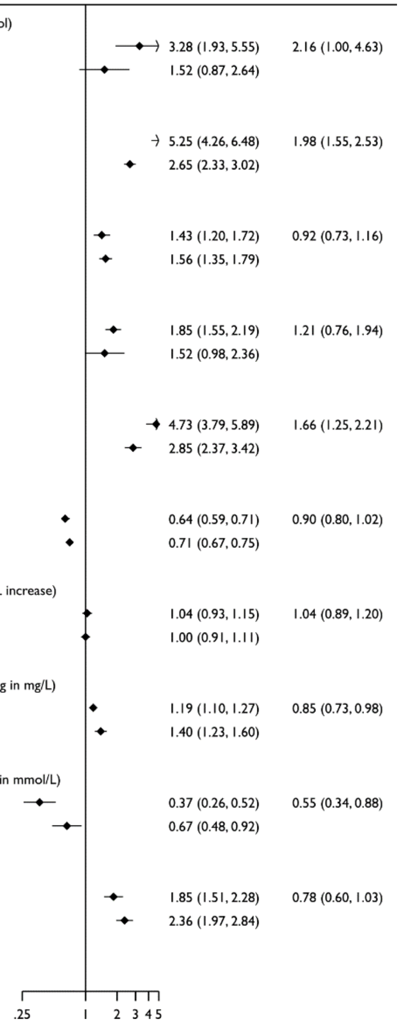

Hazard ratios are age- and sex-adjusted. The shaded diamonds indicate the hazard ratio (HR) and error bars denote the 95% confidence interval (CI) for the relation of each characteristics with the risk of death outcome. The ratio of hazard ratio (RHR) summarises the difference (HSE-SHS is the referent group) between that effect estimates for the outcome. HbA1c, glycosylated haemoglobin; FEV1, forced expiratory volume in one second; and HDL, high-density lipoprotein.

References

1. Andersson TM, Weiderpass E, Engholm G, et al. Avoidable cancer cases in the Nordic countries - The impact of overweight and obesity. *Eur J Cancer* 2017; **79**: 106-18.
2. US Department of Health Human Services. The health consequences of smoking—50 years of progress: a report of the Surgeon General. Atlanta, GA: US Department of Health and Human Services, Centers for Disease …; 2014.
3. Batty GD, Kivimaki M, Gray L, Davey Smith G, Marmot MG, Shipley MJ. Cigarette smoking and site-specific cancer mortality: testing uncertain associations using extended follow-up of the original Whitehall study. *Ann Oncol* 2008; **19**(5): 996-1002.
4. Batty GD, Kivimaki M, Bell S, et al. Psychosocial characteristics as potential predictors of suicide in adults: an overview of the evidence with new results from prospective cohort studies. *Transl Psychiatry* 2018; **8**(1): 22.
5. Fry A, Littlejohns TJ, Sudlow C, et al. Comparison of Sociodemographic and Health-Related Characteristics of UK Biobank Participants With Those of the General Population. *Am J Epidemiol* 2017; **186**(9): 1026-34.
